# Precision immuno-oncology approach for four malignant tumors in siblings with constitutional mismatch repair deficiency syndrome

**DOI:** 10.1038/s41698-024-00597-8

**Published:** 2024-05-21

**Authors:** Hana Palova, Anirban Das, Petra Pokorna, Viera Bajciova, Zdenek Pavelka, Marta Jezova, Karol Pal, Jose R. Dimayacyac, Logine Negm, Lucie Stengs, Vanessa Bianchi, Klara Vejmelkova, Kristyna Noskova, Marie Jarosova, Sona Mejstrikova, Peter Mudry, Michal Kyr, Tomas Merta, Pavel Tinka, Klara Drabova, Stefania Aulicka, Robin Jugas, Uri Tabori, Ondrej Slaby, Jaroslav Sterba

**Affiliations:** 1 Central European Institute of Technology, Masaryk University, Brno, Czech Republic; 2 Department of Biology, Faculty of Medicine, Masaryk University, Brno, Czech Republic; 3 Division of Haematology Oncology, The Hospital for Sick Children, Toronto, ON Canada; 4 Department of Paediatrics, University of Toronto, Toronto, ON Canada; 5 The Arthur and Sonia Labatt Brain Tumour Research Centre, The Hospital for Sick Children, Toronto, ON Canada; 6 Department of Biochemistry, Faculty of Science, Masaryk University, Brno, Czech Republic; 7 Department of Pediatric Oncology, University Hospital Brno, and Faculty of Medicine, ERN PaedCan Center, Masaryk University, Brno, Czech Republic; 8 Department of Pathology, University Hospital Brno and Faculty of Medicine, Masaryk University, Brno, Czech Republic; 9 Department of Pharmacology, Faculty of Medicine, Masaryk University, Brno, Czech Republic; 10 Clinical Pharmacy Section of Hospital Pharmacy, University Hospital Brno, Brno, Czech Republic; 11 Department of Internal Medicine, Hematology and Oncology, University Hospital Brno and Faculty of Medicine, Masaryk University, Brno, Czech Republic; 12 Institute of Medical Genetics and Genomics, University Hospital Brno and Faculty of Medicine, Masaryk University, Brno, Czech Republic; 13 Department of Pediatric Neurology, University Hospital Brno, and Faculty of Medicine, Masaryk University, Brno, Czech Republic

**Keywords:** Molecular medicine, Paediatric cancer, CNS cancer, Cancer genetics

## Abstract

Constitutional mismatch repair deficiency (CMMRD) is a rare syndrome characterized by an increased incidence of cancer. It is caused by biallelic germline mutations in one of the four mismatch repair genes (MMR) genes: *MLH1*, *MSH2*, *MSH6*, or *PMS2*. Accurate diagnosis accompanied by a proper molecular genetic examination plays a crucial role in cancer management and also has implications for other family members. In this report, we share the impact of the diagnosis and challenges during the clinical management of two brothers with CMMRD from a non-consanguineous family harbouring compound heterozygous variants in the *PMS2* gene. Both brothers presented with different phenotypic manifestations and cancer spectrum. Treatment involving immune checkpoint inhibitors significantly contributed to prolonged survival in both patients affected by lethal gliomas. The uniform hypermutation also allowed immune-directed treatment using nivolumab for the B-cell lymphoma, thereby limiting the intensive chemotherapy exposure in this young patient who remains at risk for subsequent malignancies.

## Introduction

Constitutional mismatch repair deficiency (CMMRD) is a rare cancer-predisposition syndrome resulting from biallelic germline mutations in one of the four mismatch repair (MMR) genes: *MLH1*, *MSH2*, *MSH6*, or *PMS2*^1^. While clinical manifestations are often nonspecific and overlap with other syndromes such as neurofibromatosis type-I^2,3^, a diagnosis in a pediatric patient can also be suspected by the presence of either synchronous or rapid-onset of specific metachronous malignancies^4,5^. These include gastrointestinal, hematological, and central nervous system cancers, most frequently high-grade gliomas^6^. Accurate diagnosis is important for cancer management and also has implications for other family members^6,7^. Cancers in CMMRD patients are refractory to agents like temozolomide and mercaptopurines which need an intact MMR machinery for their cytotoxic action, but these tumors uniformly harbor high tumor mutation (TMB) and microsatellite indel (MSI) burden, making them amenable to immune checkpoint inhibition (ICI)^8–13^. In this report, we share the impact of the diagnosis and challenges during the clinical management of two brothers with CMMRD from a non-consanguineous family harboring compound heterozygous variants in the *PMS2* gene, who presented with different phenotypic manifestations and cancer spectrum.

## Results

### Case presentation: patient 1

**Fig. 1:**
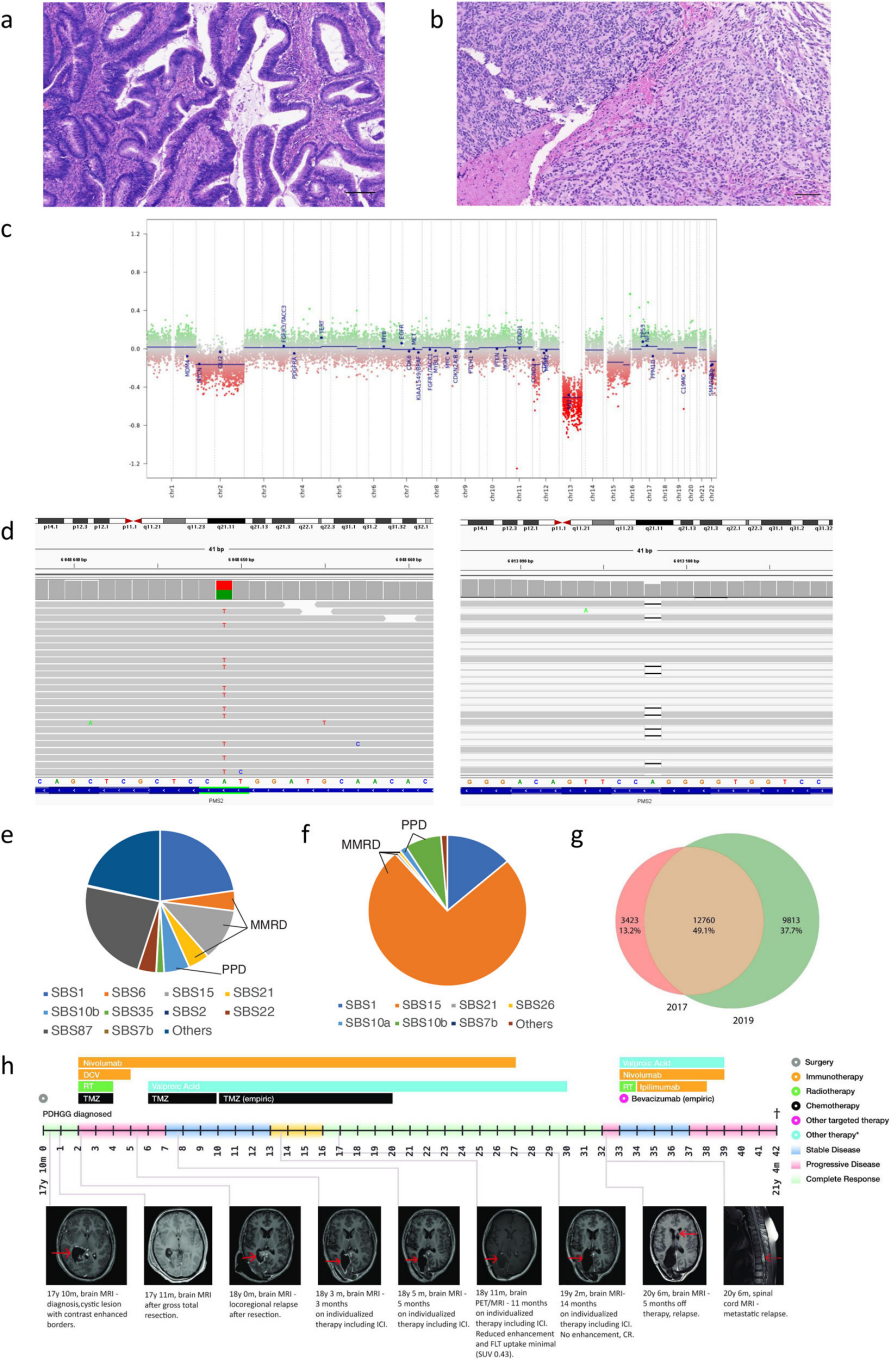
Histopathology, molecular diagnostic results, and therapy and imaging methods timeline in Patient 1. **a** Hematoxylin-eosin staining showing well-to-moderately differentiated colorectal adenocarcinoma growing into the muscularis propria. Magnification 50x, scale bar 100 µm. **b** Hematoxylin-eosin staining showing pediatric-type diffuse high-grade glioma (PDHGG) with a primitive neuronal component (left) (biopsy 2017). Magnification ×50, scale bar 100 µm. **c** Copy number variations output from MolecularNeuropathology.org classifier in PDHGG (biopsy 2019)—depiction of chromosome 1 to 22. Gains/amplifications represent positive, losses negative deviations from the baseline. Twenty-nine brain tumor relevant gene regions are highlighted for easier assessment^31^. **d** Mutations in *PMS2* c.2 T > A/p.(M1K) (left) and c.2521del/p.(W841Gfs*10) (right) identified by whole-exome sequencing visualized in The Integrative Genomics Viewer tool^32^. **e** Mutational signatures (MS) analysis in PDHGG (biopsy 2017): Signatures single-base substitution (SBS) 6, SBS15 and SBS21 associated with mismatch repair deficiency (MMRD) were identified in the tumor. SBS10b associated with polymerase-proofreading deficiency (PPD) was also identified as the result of pathogenic *POLE* mutation. **f** MS analysis in PDHGG (biopsy 2019): MMRD-related SBS15 was found to be the most represented signature. As for the PPD, SBS10b together with similar SBS10a were identified in the tumor. **g** Mutational overlap in PDHGG 2017 and 2019 biopsy. **h** Schematic overview of patient’s individualized treatment plan including immune checkpoint inhibitors. Overview of imaging tests and treatment responses in patient. Abbreviations in the figure: DCV dendritic cell vaccination, RT radiotherapy, TMZ temozolomide, MRI magnetic resonance imaging, ICI immune checkpoint inhibitor, PET positron emission tomography, FLT ^18^F-fluorothymidine, SUV standardized uptake value, CR complete response, Other therapy* other potentially anticancer therapy.

The index patient was diagnosed with colon adenocarcinoma at 14-years (Fig. 1a; Fig. S1), achieved complete remission following hemicolectomy and standard FOLFOX chemotherapy, and subsequently developed a pediatric-type diffuse high-grade glioma (PDHGG; histone and IDH-wild type with chromosome 13q loss), 4-years later (Fig. 1b, c). There was no consanguinity or history of cancers in the family, as well as no neuro-cutaneous stigmata. Germline sequencing confirmed CMMRD caused by distinct *PMS2* pathogenic variants – c.2 T > A/p.(M1K) and c.2521del/p.(W841Gfs*10), each inherited from asymptomatic parents (Fig. 1d). Both cancers were hypermutated with mutational signatures of MMR deficiency^14,15^ (Fig.1 e, f), loss of PMS2 expression on immunohistochemistry (IHC) and high exomic MSI burden (colon: 207; PDHGG: 1360; median for MMR-proficient controls: 17). Additionally, the PDHGG harbored extreme tumor mutational burden (TMB) (337.56 mut/Mb) likely contributed by a somatic, pathogenic *POLE* driver mutation in the exonuclease domain (p.E978G)^16,17^ accompanied by COSMIC single-base substitution signature 10^14,15^. Following focal radiation, the child was enrolled in a clinical trial of a dendritic cell vaccine (5 doses)^18^ and then started on nivolumab. Temozolomide, initially initiated, was stopped following multi-disciplinary discussions^1^. The child achieved complete remission, and nivolumab was continued for 24 months. Ongoing surveillance identified no new primary cancers.

Unfortunately, a disseminated recurrence 5-months after stopping nivolumab was confirmed as hypermutant and MSI-high PDHGG (TMB: 461.87 mut/Mb; exomic MSI burden: 2101) with the same *POLE* mutation but an otherwise evolved mutational spectrum from the original biopsy (Fig. 1g). The recurrent tumor did not show any known mechanisms of immune evasion, including either new pathogenic variants in the JAK/STAT pathway or loss of heterozygosity of HLA alleles. In contrast, the paired gene analysis for neoantigen expression suggested retained immunogenicity (Supplementary Fig. 1 in the Supplementary Data). Focal radiation was administered to sites of disease recurrence in combination with bevacizumab, and nivolumab was reinitiated. Ipilimumab was added post-radiation, but the treatment had to be interrupted for thrombocytopenia^19^. Autoimmune toxicity and concomitant hematological malignancy were excluded by exhaustive investigations. Ultimately, the thrombocytopenia was attributed to a Parvovirus-B19 infection, and the child was treated with intravenous immunoglobulin. However, during this period, he presented with status epilepticus. Magnetic resonance imaging (MRI) demonstrated disseminated glioma progression (Fig. 1h). He was started on dexamethasone and shifted to palliative care, leading to demise 43 months from his PDHGG diagnosis.

### Case presentation: patient 2

**Fig. 2:**
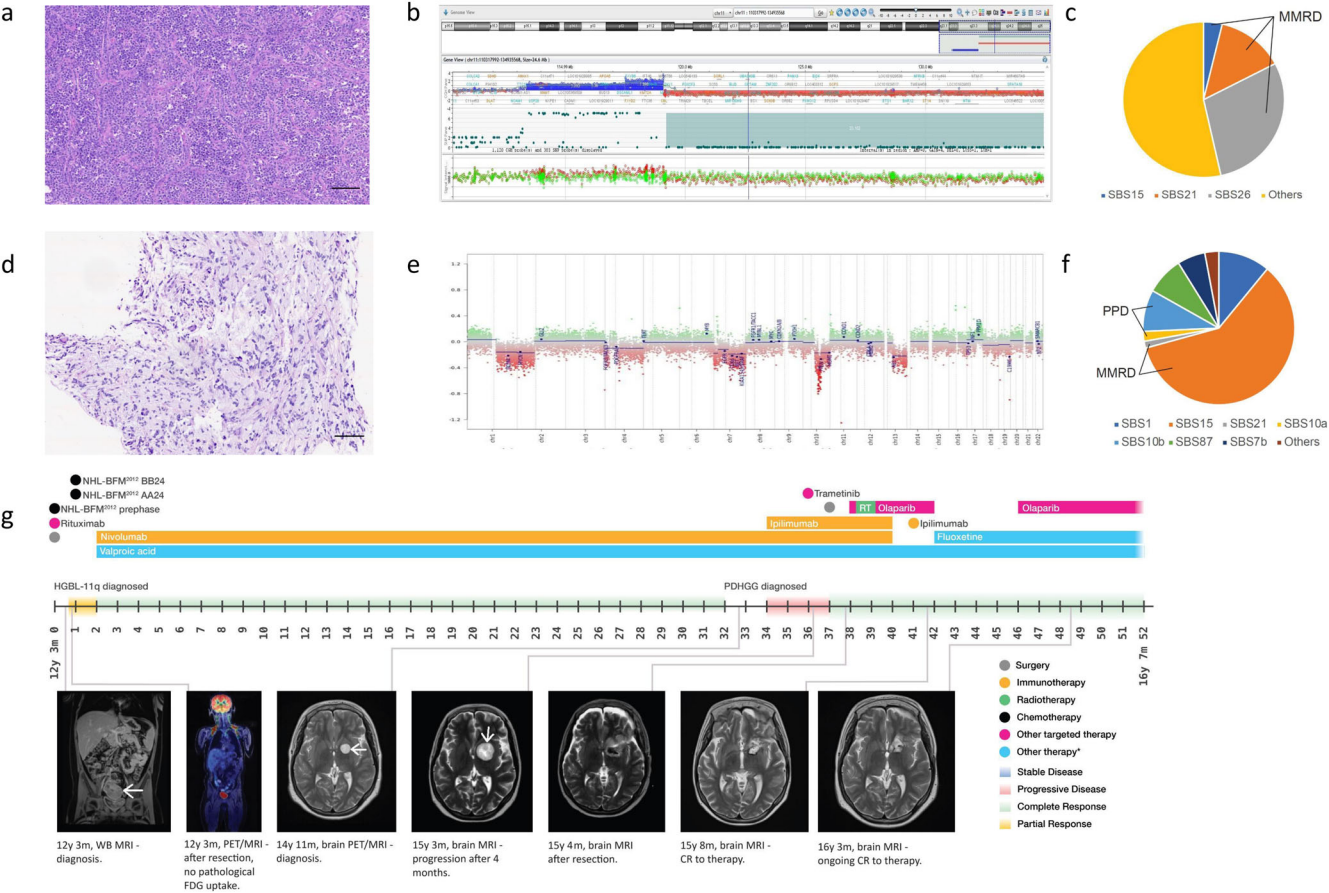
Histopathology, molecular diagnostic results, and therapy and imaging methods timeline in Patient 2. **a** Hematoxylin-eosin staining showing medium-sized blastic cells and starry sky pattern (Burkitt-like morphology, *MYC*-negative) in patient’s high-grade B-cell lymphoma with 11q aberrations (HGBL-11q). Magnification ×50, scale bar 100 µm. **b** Chromosome 11q aberrations (gain/loss) identified in patient’s HGBL-11q using array-CGH visualized in CytoGenomics software (Agilent Technologies, CA, USA). **c** Mutational signatures (MS) analysis in HGBL-11q: Signatures single-base substitution (SBS) 15, SBS21 and SBS26 associated with mismatch repair deficiency (MMRD) were identified in the tumor. **d** Hematoxylin-eosin staining showing bizarre multinucleated cells in patient’s pediatric-type diffuse high-grade glioma (PDHGG). Magnification ×50, scale bar 100 µm. **e** Copy number variations output from MolecularNeuropathology.org classifier in the patient’s PDHGG – depiction of chromosome 1 to 22. Gains/amplifications represent positive, losses negative deviations from the baseline. Twenty-nine brain tumor relevant gene regions are highlighted for easier assessment^31^. **f** MS analysis in PDHGG: MMRD-related SB15, as the most represented signature, together with SBS21 were identified in the tumor. SBS10a and SBS10b associated with polymerase-proofreading deficiency (PPD) were also identified as the result of pathogenic *POLE* mutation. **g** Schematic overview of patient’s individualized treatment plan including immune checkpoint inhibitors. Overview of imaging tests and treatment responses in patient. Abbreviations in the figure: NHL-BFM non-Hodgkin lymphoma Berlin-Frankfurt-Münster, RT radiotherapy, WB MRI whole-body magnetic resonance imaging, HGBL-11q high-grade B-cell lymphoma with 11q aberrations, PET/MRI positron emission tomography/magnetic resonance imaging, FDG F-fluorodeoxyglucose, PDHGG pediatric-type diffuse high-grade glioma, Other therapy* other potentially anticancer therapy.

The brother of the index patient was diagnosed with CMMRD as part of cascade testing and initiated on the published cancer surveillance protocol^6^. Notably, he also harbored café-au-lait macules. Surveillance imaging detected asymptomatic abdominal lymphadenopathy. A biopsy confirmed high-grade mature B-cell-lymphoma with 11q aberration but lacking *MYC*/8q24 rearrangement^20,21^ (Fig. 2a, b). The patient received two courses of intensive chemotherapy as per the NHL-BFM 2012 protocol, along with rituximab. Hypermutation (TMB: 107.74 mut/Mb) driven by MMR deficiency mutational signatures (COSMIC)^14,15^ (Fig. 2c), loss of PMS2 protein expression, and high exomic MSI burden (1830) were detected. Chemotherapy was stopped, and the patient was treated with nivolumab following a multi-disciplinary consensus. With sustained remission lasting >24 months, a decision to taper nivolumab was made. After 9 months on this tapering schedule at a dose of 1 mg/kg q2-weeks, the surveillance MRI revealed a lesion in the putamen/globus pallidus. A biopsy confirmed this as PDHGG with losses in chromosomes 1q, 2p, 4, 7, 10q, 13q, and extreme TMB (142 mut/Mb) with a driver mutation in the *POLE* exonuclease domain (p.V411L)^16,17^, corresponding mutational signatures and high exomic MSI burden (534) (Fig. 2f). The patient was treated with tumor debulking, re-irradiation, and re-initiation of full-dose nivolumab (3 mg/kg q2-weeks) (Fig. 2g). Initial radiological progression led to a decision to add trametinib, as the tumor harbored a truncating *NF1* mutation (p.G824*) and previous reports of success using this approach^22^. However, trametinib had to be withheld following significant cardiac toxicity. Ipilimumab was then added to nivolumab, and complete remission was achieved. Unfortunately, ICI treatment had to be stopped due to symptomatic autoimmune pulmonary toxicity. Infectious etiologies were excluded, and rapid clinical and radiological improvements were noted after initiating steroids. As autoimmune pneumonitis is more frequently reported with nivolumab, a decision was made to rechallenge the patient with ipilimumab monotherapy^23^. However, this, too, had to be stopped following severe hepato-toxicity. The patient is currently on imaging surveillance without evidence of any cancer, 13 months after stopping ICI treatment, >23 months since PDHGG diagnosis, and 5 years from the lymphoma diagnosis.

## Discussion

We gained several important clinical insights during the management of these two siblings with CMMRD, which, we believe, will be extremely useful in managing future patients with such rare and complex disorders.

First, we observed that the clinical phenotype can vary between two siblings harboring the same germline pathogenic variants in CMMRD. Here, only the younger brother harbored café-au-lait macules, which is otherwise the commonest clinical manifestation triggering evaluation for germline disorders like neurofibromatosis and CMMRD in the context of a childhood malignancy^2,3^. It was recently demonstrated that children with CMMRD and café-au-lait macules uniformly lack germline variants in *NF1* or *SPRED1* genes^24^ but can harbor somatic mosaicism for such aberrations explaining such inter-patient heterogeneity^25^. Second, the high prevalence and early onset of multiple malignancies in this aggressive cancer syndrome highlight the importance of systematic cancer surveillance in these patients^6,8^. Notably, both malignancies in the second sibling were detected on imaging-based surveillance in the asymptomatic state, plausibly contributing to the improved outcome in this patient.

Next, we demonstrate that the germline genomic instability in CMMRD patients not only leads to the development of hypermutant cancers but can also contribute to the rapid evolution of the mutational spectrum between the primary diagnosis and recurrence. The first patient’s PDHGG harbored a combination of MMRD and polymerase-proofreading deficiencies, which not only contributed to the extreme TMB^16,17^ and MSI burden both at diagnosis and recurrence but resulted in the acquisition of diverse novel somatic variants at recurrence. This underscores that a repeat biopsy followed by genomic analyses of a recurrent tumor should be an important consideration in a patient with CMMRD. As recently reported for these genomically unstable RRD cancers^10,26^, known drivers of immune evasion in lung and other cancers, including defects in antigen presentation and interferon signaling, were not demonstrated to be enriched at recurrence and persistently high immunogenicity was noted. This underscores that a repeat biopsy followed by genomic analyses of a recurrent tumor should be an important consideration in a patient with CMMRD, as this can allow re-initiation of treatment and continuation of immunotherapy can lead to second, even if, delayed responses^10,26^.

Last, while the impact of ICI treatment was reiterated for deadly gliomas with prolonged survival in both patients, the uniform hypermutation also allowed immune-directed treatment using nivolumab for the B-cell lymphoma, thereby limiting intensive chemotherapy exposure in this young patient who remains at risk for subsequent malignancies^8^. This is in contrast to T-cell lymphomas, where ICI treatment may not only be ineffective but can add to risk of disease progression^9,27^. Importantly following the success in selected young patients with favorable biomarkers^26^ including high TMB, MSI and immune infiltrates, by using post-resection ICI treatment and avoiding chemo-radiation^12^, this approach has generated enthusiasm as an upcoming clinical trial. However, we also note that there may be a need for sustained immune surveillance in these patients with germline genomic instability, as interruption of immunotherapy was clinically correlated with recurrence/progression at multiple time points in both patients. This can be challenging with the use of combinatorial ICI-based treatments, like anti-CTLA4 and anti-PD1, especially in CMMRD patients where even non-malignant cells in the body accumulate mutations and MS-indels at high rates, leading to high rates of autoimmune toxicities in a recently published study, was also seen in our patients^19,26^. Hence, while combinatorial strategies can be effective salvage options upon failure of checkpoint-inhibitor monotherapy, it can be challenging to continuously deliver existing combinations in CMMRD patients. Novel treatment regimens need to be explored to balance efficacy while minimizing toxicity to improve the patient’s tolerance to immune-based combinations. Immune surveillance using vaccines targeting tumor neoantigens are in clinical trials in patients with heterozygous MMR-deficient Lynch syndrome following success in preclinical models^28,29^, and need to be systematically explored for patients with CMMRD.

Limited data exist on the optimal duration of immunotherapy for patients with CMMRD and for children in particular. The ICI treatment duration for the first patient was 24 months, which at that time was considered as standard of care for other cancers. For the second brother we have tried to keep him on prolonged immune surveillance while trying to decrease the risk of side effects with lower dose. The question if the full dose of nivolumab would prevent evolution of the secondary glioblastoma remains unanswered.

We conclude that, despite being a highly aggressive cancer syndrome, a timely diagnosis of CMMRD and monitoring of affected patients according to established algorithms is essential for the early detection of malignancy as this can be associated with longer patient survival. Comprehensive molecular testing is paramount for developing individualized treatment plans in affected patients. While immunotherapy plays an indispensable role in the treatment of CMMRD-driven tumors, novel regimens are urgently needed to maintain sustained systemic immune surveillance while limiting treatment toxicity.

## Methods

### Whole-exome sequencing

Whole-exome sequencing (WES) using TruSeq DNA Exome Kit, NextSeq 500/550 Mid Output Kit, and NextSeq 500 device (all Illumina, CA, USA) was done in both brothers’ tumors. 100 ng of DNA obtained from formalin-fixed paraffin-embedded (FFPE) tumor samples served as the input material for the somatic (cancer) exome. 400 ng of DNA obtained from leukocytes of peripheral blood was used as the input material for the germline exome. All steps above were performed according to the manufacturer’s protocol. WES was performed with high coverage, where at least 90% of targeted regions were covered at least 20 times. This was followed by bioinformatic analysis using a standardized pipeline.

A spectrum of novel and described variants affecting both oncogenes and tumor suppressor genes were identified in both patients’ tumors and selected variants are listed in Table 1a–d.

**Table 1a:** Selected somatic variants identified in Patient 1’s pediatric-type diffuse high-grade glioma (PDHGG) using whole exome sequencing (WES)

Gene	Protein (according to Uniprot)	Variant (c.DNA/protein)	dbSNP identifier	Biopsy
*PIK3CA*	Phosphatidylinositol 4,5-bisphosphate 3-kinase catalytic subunit alpha isoform	c.1360 G > T/p.D454Y	-	2017, 2019
*PIK3CA*	“	c.2422 C > T/p.R808W	rs200671228	2017, 2019
*PIK3R1*	Phosphatidylinositol 3-kinase regulatory subunit alpha	c.1126 G > A/p.G376R	rs1057519757	2017, 2019
*PIK3R1*	“	c.1507 C > T/p.R503W	-	2017, 2019
*PDGFRA*	Platelet-derived growth factor receptor alpha	c.1715 A > C/p.Y572S	-	2017, 2019
*PDGFRA*	“	c.3265 C > A/ p.L1089M	-	2017, 2019
*RET*	Proto-oncogene tyrosine-protein kinase receptor Ret	c.2437 C > T/p.R813W	rs779996040	2017, 2019
*TP53*	Cellular tumor antigen p53	c.844 C > T/p.R282W	rs28934574	2017, 2019
*NF1*	Neurofibromin	c.3574 G > T/p.E1192*	-	2017, 2019
*NF1*	“	c.4600 C > T/p.R1534*	rs760703505	2017, 2019
*NF1*	“	c.6951 G > A/p.W2317*	-	2017, 2019
*POLE*	DNA polymerase epsilon catalytic subunit A	c.5278 G > A/p.V1760M	rs373272795	2017, 2019
*POLE*	“	c.3697 C > T/p.R1233*	rs745750549	2019
*NF1*	Neurofibromin	c.2033dup/p.I679fs	rs1232596244	2019
*RAD50*	DNA repair protein RAD50	c.3598 C > T/p.R1200	rs750586158	2019

**Table 1b:** Selected somatic variants identified in Patient 1’s colorectal carcinoma using WES

Gene	Protein (according to Uniprot)	Variant (c.DNA/protein)	dbSNP identifier
*KRAS*	GTPase KRas	c.35 G > C/p.G12A	rs121913529
*PIK3CA*	Phosphatidylinositol 4,5-bisphosphate 3-kinase catalytic subunit alpha isoform	c.1634 A > G/p.E545G	rs121913274
*RET*	Proto-oncogene tyrosine-protein kinase receptor Ret	c.2099 T > C/p.M700T	-
*BRAF*	Serine/threonine-protein kinase B-raf	c.1208dup/p.A404fs	rs777474487
*AMER1*	APC membrane recruitment protein 1	c.1489 C > T/p.R497*	-
*APC*	Adenomatous polyposis coli protein	c.2563 G > T/p.E855*	-
*APC*	“	c.4666dup/p.T1556fs	-
*EP300*	Histone acetyltransferase p300	c.6329_6330insT/p.Q2110fs	-
*EP300*	“	c.6316del/p.M2106fs	-
*BARD1*	BRCA1-associated RING domain protein 1	c.672dup/p.E225fs	-
*BARD1*	“	c.623dup/p.K209fs	rs587780033
*SMAD4*	Mothers against decapentaplegic homolog 4	c.153dup/p.D52fs	-
*SMAD4*	“	c.1082 G > A/p.R361H	rs377767347
*TP53*	Cellular tumor antigen p53	c.718 A > C/p.S240R	-
*POLE*	DNA polymerase epsilon catalytic subunit A	c.875 A > G/p.Q292R	-

**Table 1c:** Selected somatic variants identified in Patient 2’s high-grade B-cell lymphoma with 11q aberrations (HGBL-11q)

Gene	Protein (according to Uniprot)	Variant (c.DNA/protein)	dbSNP identifier
*TP53*	Cellular tumor antigen p53	c.821 T > C/p.V274A	rs1057520006
*TP53*	“	c.503 A > G/p.H168R	rs867114783
*ATM*	Serine-protein kinase ATM	c.640del/p.S214fs	rs1388051413
*CCND3*	G1/S-specific cyclin-D3	c.811dup/p.R271fs	-
*PTEN*	Phosphatidylinositol 3,4,5-trisphosphate 3-phosphatase and dual-specificity protein phosphatase PTEN	c.521 A > G/p.Y174C	rs864622341
*ROS1*	Proto-oncogene tyrosine-protein kinase ROS	c.3989 A > T/p.N1330I	-
*ROS1*	“	c.2122 T > C/p.S708P	-
*DDX3X*	ATP-dependent RNA helicase DDX3X	c.1415 A > G/p.H472R	-
*FAT4*	Protocadherin Fat 4	c.1397 A > G/p.N466S	-
*FAT4*	“	c.1849A>C/p.T617P	-
*FAT4*	“	c.3656 T > C/p.I1219T	-
*FAT4*	“	c.8606 A > G/p.D2869G	-
*FAT4*	“	c.11181 C > A/p.F3727L	-
*FAT4*	“	c.14804 T > C/p.L4935P	-
*NFRKB*	Nuclear factor related to kappa-B-binding protein	c.1007del/p.K336fs	-
*LRP1B*	Low-density lipoprotein receptor-related protein 1B	c.12715 T > C/p.C4239R	-
*LRP1B*	“	c.12130 A > T/p.T4044S	-
*LRP1B*	“	c.11908 A > G/p.R3970G	rs970306366
*LRP1B*	“	c.8831 A > T/p.D2944V	-
*LRP1B*	“	c.7927 T > G/p.F2643V	-
*LRP1B*	“	c.6940 A > G/p.M2314V	rs1031301423
*LRP1B*	“	c.4025 T > A/p.V1342D	-

In addition to TP53 and CCND3, other genes found to be frequently mutated in Burkitt lymphoma and HGBL-11q has been included (DDX3X, FAT4, NFRKB, LRP1B)^1^

**Table 1d:** Selected somatic variants identified in Patient 2’s PDHGG using WES

Gene	Protein (according to Uniprot)	Variant (c.DNA/protein)	dbSNP identifier
*ATM*	Serine-protein kinase ATM	c.8287 C > T/p.R2763*	rs876659872
*NF1*	Neurofibromin	c.2470 G > T/p.G824*	-
*BRCA1*	Breast cancer type 1 susceptibility protein	c.2990dup/p.N997fs	-
*BRIP1*	Fanconi anemia group J protein	c.752 G > A/p.R251H	rs780834054
*TP53*	Cellular tumor antigen p53	c.981 T > G/p.Y327*	rs879254077
*TP53*	Cellular tumor antigen p53	c.707 A > G/p.Y236C	rs730882026
*TP53*	Cellular tumor antigen p53	c.541 C > T/p.R181C	rs587782596
*TP53*	Cellular tumor antigen p53	c.523 C > T/p.R175C	rs138729528
*POLE*	DNA polymerase epsilon catalytic subunit A	c.1231 G > T/p.V411L	rs1057519945
*ATRX*	Transcriptional regulator ATRX	c.6332 G > A/p.R2111Q	-
*KMT2A*	Histone-lysine N-methyltransferase 2A	c.8095 C > T/p.R2699*	rs587783680
*BLM*	RecQ-like DNA helicase BLM	c.3670 G > T/p.E1224*	-
*APC*	Adenomatous polyposis coli protein	c.8446 C > T/p.R2816*	-
*ARID1A*	AT-rich interactive domain-containing protein 1A	c.3667 C > T/p.R1223C	rs1227227387
*CHEK2*	Serine/threonine-protein kinase Chk2	c.823 G > A/p.E275K	-
*TSC1*	Hamartin	c.518 C > T/p.A173V	rs777484049
*ROS1*	Proto-oncogene tyrosine-protein kinase ROS	c.4583dup/p.N1528fs	rs776274768
*RET*	Proto-oncogene tyrosine-protein kinase receptor Ret	c.2437 C > T/p.R813W	rs779996040

### Determination of TMB and mutational signatures

The TMB (single nucleotide variants per megabase) from WES data was calculated by counting total number of somatic single nucleotide variants divided by total number of callable bases in megabases (~50 Mb). Mutational signatures were examined according to COSMIC version 3.2 (Figs.1e, f and 2c, f)^15^.

### Microsatellite indel calling

The methodology was previously described in Das et al.^9^. Microsatellite indels were called on the bam files of tumor and matched normal samples, using an in-house pipeline using MSMuTect v1. The detailed methods for this algorithm have been previously reported^30^.

### RNA sequencing: gene expression profiling

In all Patient 1’s tumors (CRC, both PDHGGs) and Patient 2’s tumors (HGBL-11q and PDHGG), gene expression profiling using RNA sequencing was performed. Messenger RNA was purified from 300 ng of RNA extracted from frozen tumor tissue using NEBNext Poly(A) mRNA Magnetic Isolation Module (New England Biolabs, MA, USA). Sequencing libraries from polyA selected mRNA were prepared using NEBNext Ultra II Directional RNA Library Prep Kit (New England Biolabs) and sequenced on the NextSeq 500 device using NextSeq 500/550 Mid Output Kit v2,5 (75 cycles) (Illumina), all according to the manufacturer’s recommendations. Raw sequencing reads were quality-checked with FastQC and then aligned against GRCh37. The gene counts were estimated with Gencode gene definitions and compared with reference gene counts from the GTEx database of non-disease tissue-specific samples. The gene counts were TMM normalized, and a t-test statistic was used. For selected genes (211 genes), which were classified according to the pathways they participate in, the fold change value ≤ 0.5 was recorded and graphically expressed (+ to ++++++). Selected gene expressions are shown in the Supplementary Data, Supplementary Tables 2 and 3.

### Additional analyses of WES and RNA sequencing data

Additional analyses of WES and RNA sequencing data were performed. Description and results of these analyses can be found in the Supplementary Data.

### Targeted RNA sequencing: fusion genes examination

Examination of fusion genes using RNA sequencing was performed in both brothers and all the respective tumors. Sequencing libraries were prepared using TruSight RNA Pan-Cancer Panel, loaded onto NextSeq 500/550 Mid Output Kit v2,5 (150 cycles) cartridge, and sequenced using NextSeq 500 sequencing device (all Illumina, CA, USA), all according to manufacturer’s recommendations. As the input material, 50 ng of RNA isolated from a frozen tumor was used. A parablock was obtained from this frozen tumor tissue, and the content of the tumor component was determined by a pathologist. Sequencing data were bioinformatically processed using a standardized pipeline.

No fusion genes were detected in any of the samples, and the absence of *MYC* fusion was confirmed in Patient 2’s lymphoma.

### Methylation profiling

Methylation profiling was done in both patients’ tumors of central nervous system (CNS) using 850k EPIC Methylation Array Kit and NextSeq 550 sequencing device (all Illumina, CA, USA) according to manufacturer’s protocol. As the input material, 250 ng of DNA isolated from frozen tissue samples was used. DNA methylation-based classification of CNS tumors was performed according to Capper et al.^31^.

Both CNS tumors were classified as diffuse pediatric-type HGG, RTK1 subtype, subclass A, which corresponds to the methylation class characteristics, stating that tumors developing in the context of CMMRD predominantly belong to this category. In addition to tumor classification, the output of the analysis was also a copy number variation profile (Fig.1c, 2e) and determination of methylguanine-DNA-methyltransferase promotor methylation status (both tumors resulted as unmethylated).

### Microarray-based comparative genomic hybridization (array-CGH)

Oligonucleotide array-CGH was performed using SurePrint G3 Cancer CGH + SNP Microarray Kit, 4x180K (Agilent Technologies, CA, USA). Sample DNA and sex-matched control DNA (Human Genomic DNA, Agilent) were restricted, labeled, and purified with SureTag Complete DNA Labeling Kit according to manufacture protocol (Agilent). The quality of DNA was checked by Nanodrop, and the specific activity of both DNA, sample, and reference were in an optimal range. Hybridization was done with Oligo aCGH/ChIP-Chip Hybridization Kit (Agilent) and ran for 24 hours at 67 °C. Analysis was performed according to the protocol provided by the supplier (Agilent Oligonucleotide Array-Based CGH for Genomic DNA Analysis). Arrays were scanned using a SureScan High Resolution Microarray Scanner (Agilent). Data were imported using the Feature Extraction V.5.0.2.1 software, and results were analyzed using CytoGenomics software v5.1.2.1 (Agilent). All genomic coordinates were established using the human reference genome GRCh38. Detected aberrations in both patients’ tumor are shown in the Supplementary Data.

### Reporting summary

Further information on research design is available in the Nature Research Reporting Summary linked to this article.

### Supplementary information

Reporting Summary

Supplementary data

## Data Availability

The raw sequencing and array-CGH data are not publicly available due to data privacy regulations and restrictions for use of such data, as stated in the study protocol and patient consent form. Data supporting the findings of this study are available within the article and its supplementary data files.

## Code availability

Bioinformatics pipeline code is available upon request.
