# Supplementary material for: Precision immuno-oncology approach for four malignant tumors in siblings with constitutional mismatch repair deficiency syndrome
Source: NPJ Precis Oncol. 2024 May 21;8:110. doi: 10.1038/s41698-024-00597-8 (PMC11109258; doi:10.1038/s41698-024-00597-8)
Supplement: Supplementary file 1 — Reporting Summary [file 41698_2024_597_MOESM1_ESM.pdf]

nature portfolio

Corresponding author(s): Jaroslav Sterba
Ondrej Slaby

Last updated by author(s): Jan 26, 2024

Reporting Summary

Nature Portfolio wishes to improve the reproducibility of the work that we publish. This form provides structure for consistency and transparency in reporting. For further information on Nature Portfolio policies, see our Editorial Policies and the Editorial Policy Checklist.

Statistics

For all statistical analyses, confirm that the following items are present in the figure legend, table legend, main text, or Methods section.

n/a	Confirmed	
☒	☐	The exact sample size (n) for each experimental group/condition, given as a discrete number and unit of measurement
☒	☐	A statement on whether measurements were taken from distinct samples or whether the same sample was measured repeatedly
☒	☐	The statistical test(s) used AND whether they are one- or two-sided<br>*Only common tests should be described solely by name; describe more complex techniques in the Methods section.*
☒	☐	A description of all covariates tested
☒	☐	A description of any assumptions or corrections, such as tests of normality and adjustment for multiple comparisons
☒	☐	A full description of the statistical parameters including central tendency (e.g. means) or other basic estimates (e.g. regression coefficient) AND variation (e.g. standard deviation) or associated estimates of uncertainty (e.g. confidence intervals)
☒	☐	For null hypothesis testing, the test statistic (e.g. F, t, r) with confidence intervals, effect sizes, degrees of freedom and P value noted<br>*Give P values as exact values whenever suitable.*
☒	☐	For Bayesian analysis, information on the choice of priors and Markov chain Monte Carlo settings
☒	☐	For hierarchical and complex designs, identification of the appropriate level for tests and full reporting of outcomes
☒	☐	Estimates of effect sizes (e.g. Cohen's d, Pearson's r), indicating how they were calculated

Our web collection on statistics for biologists contains articles on many of the points above.

Software and code

Policy information about availability of computer code

Data collection	No software was used for data collection.
Data analysis	Whole-exome sequencing: ...<br>Targeted RNA sequencing: GRCh38 alignment with STAR version 2.7.3a, QC with QualiMap version 2.2.2 and Picard version 2.27.1, fusion calling with Arriba version 2.4.0 and STARfusion version 1.12<br>RNA sequencing: GRCh37 alignment with Rsubread version 2.14.2, gene count estimate with GENCODE v19, comparison to reference dataset from GTEX v7, TMM normalisation<br>Methylation profiling: automated data analysis via MolecularNeuropathology.org platform - brain classifier v12.5 (available at https://www.molecularneuropathology.org/mnp/)<br>Array CGH: automated data analysis via CytoGenomics Analysis software v5.1.2.1 (Agilent Technologies)

For manuscripts utilizing custom algorithms or software that are central to the research but not yet described in published literature, software must be made available to editors and reviewers. We strongly encourage code deposition in a community repository (e.g. GitHub). See the Nature Portfolio guidelines for submitting code & software for further information.

Data

Policy information about availability of data

All manuscripts must include a data availability statement. This statement should provide the following information, where applicable:

- Accession codes, unique identifiers, or web links for publicly available datasets
- A description of any restrictions on data availability
- For clinical datasets or third party data, please ensure that the statement adheres to our policy

Anonymized raw sequencing data can be provided upon reasonable request send to the corresponding author after a signed data transfer agreement.

Research involving human participants, their data, or biological material

Policy information about studies with human participants or human data. See also policy information about sex, gender (identity/presentation), and sexual orientation and race, ethnicity and racism.

Reporting on sex and gender	It is a report of two siblings - brothers, no other information concerning gender and/or sexual orientation was collected as it was not relevant.
Reporting on race, ethnicity, or other socially relevant groupings	These data were not relevant for our case study, therefore they were not collected/reported.
Population characteristics	Not relevant. It is description of two distinct cases.
Recruitment	Patients were selected based on a rare medical case.
Ethics oversight	Ethics Committee of Masaryk University (26/2019), Ethics Committee of University Hospital Brno (16-120619/EK)

Note that full information on the approval of the study protocol must also be provided in the manuscript.

Field-specific reporting

Please select the one below that is the best fit for your research. If you are not sure, read the appropriate sections before making your selection.

☒ Life sciences ☐ Behavioural & social sciences ☐ Ecological, evolutionary & environmental sciences

For a reference copy of the document with all sections, see nature.com/documents/nr-reporting-summary-flat.pdf

Life sciences study design

All studies must disclose on these points even when the disclosure is negative.

Sample size	Samples were included based on the number of biopsies performed in the two patients, formal sample size calculation was not relevant to the case report.
Data exclusions	None.
Replication	Not applicable.
Randomization	Not applicable.
Blinding	Not applicable.

Reporting for specific materials, systems and methods

We require information from authors about some types of materials, experimental systems and methods used in many studies. Here, indicate whether each material, system or method listed is relevant to your study. If you are not sure if a list item applies to your research, read the appropriate section before selecting a response.

Materials & experimental systems

n/a	Involved in the study	
☒	☐	Antibodies
☒	☐	Eukaryotic cell lines
☒	☐	Palaeontology and archaeology
☒	☐	Animals and other organisms
☐	☒	Clinical data
☒	☐	Dual use research of concern
☒	☐	Plants

Methods

n/a	Involved in the study	
☒	☐	ChIP-seq
☒	☐	Flow cytometry
☒	☐	MRI-based neuroimaging

Clinical data

Policy information about clinical studies

All manuscripts should comply with the ICMJE guidelines for publication of clinical research and a completed CONSORT checklist must be included with all submissions.

Clinical trial registration	Not applicable.
Study protocol	Not applicable.
Data collection	clinical records of the University Hospital Brno, CZ, 2014 - 2023, approved by ethics commitee
Outcomes	Not applicable.

Plants

Seed stocks	Not applicable.
Novel plant genotypes	Not applicable.
Authentication	Not applicable.
