# Supplementary material for: Precision immuno-oncology approach for four malignant tumors in siblings with constitutional mismatch repair deficiency syndrome
Source: NPJ Precis Oncol. 2024 May 21;8:110. doi: 10.1038/s41698-024-00597-8 (PMC11109258; doi:10.1038/s41698-024-00597-8)
Supplement: Supplementary file 2 — Supplementary data [file 41698_2024_597_MOESM2_ESM.pdf]

SUPPLEMENTARY DATA

1. Additional analyses of whole-exome sequencing (WES) and RNA sequencing data

1.1 HLA-typing

The methodology was previously described in Das *et al* (1). Paired end fastq files from matched germline WES data were used as input to computationally determine HLA (human leukocyte antigen) Class-I types for 39 tumors using a consensus of HLAminer(2), and HLAVBSeq(3), as described and validated before (4). The top 6 HLA-types were used as input for neoantigen calling as described below.

1.2 Neoantigen calling

The methodology was previously described in Das *et al* (1). The Mutect2 vcfs generated for each tumor (described above) were used as input along with bioinformatically generated HLA-types (above) for MuPeXI (5), to get a list of strong binding candidate neoantigens per HLA-type. This uses netMHCpan (6) (to calculate all variant peptides ranging from 8–12 mer, and total candidate neoantigens was determined by selecting all neoantigens that showed '%rank'<0.5 binding affinity, denoting all strong binders (as recommended by the netMHCpan user manual). It is to be noted that all candidate neoantigens were restricted to class-I MHC proteins only.

1.3 Neoantigens from coding microsatellites

The methodology was previously described in Das *et al* (1). Microsatellite indels were called using MSMuTect v1.0 (7). The Indels were annotated using the Ensembl Variant Effect Predictor71 (VEP release/104.2). Neoantigens were identified using pVAC-seq78 software suite. Using NetMHCpan4.1 (8) algorithm (included in pVAC-seq), we predicted 8- and 9-mer neoantigens with strong binding affinity (score ≤ 500 nM) to the patients' HLA class I (A, B, or C).

1.4 Analysis of mechanisms related to immune evasion in Patient 1

1.4.1 HLA-typing

Analysis of germline WES data revealed that both brothers have the same HLA-A alleles: HLA-A31:01 and HLA-A02:01. Both of these alleles were also detected in patients' tumors. Therefore, we haven't identified evidence of loss of heterozygosity in HLA alleles from WES data.

1.4.2 Detection of variants in genes related to immune evasion

Analysis of JAK/STAT pathway genes including *JAK1, STAT1, IFNGR1, JAK2, STAT5A, STAT5B and TYK2* revealed a previously unreported nonsense variant c.3163C>T/p.Q1055* in the *JAK1* gene which was detected at both time-points. Variant has not been described and functionally characterized before, therefore its effect on protein function is not known. However, the presence of this mutation did not impact clinical response at initial treatment. Variant frequency in the initial biopsy (2017) is 22% and 14% in the recurrence (2019), suggesting this variant has no role in immune evasion.

1.4.3 Neoantigen analysis

Candidate neoantigens were identified by combined analysis of WES and RNA sequencing data. 3575 immunogenic neoantigens in the 2017 biopsy and 4871 in the 2019 biopsy were identified demonstrating that many more neoantigens are present in the later sample. Focusing on the 100 most immunogenic neoantigens shared in both biopsies and their expression comparison revealed that no overall expression change pattern was identified between biopsies 2017 and 2019 as showed in the following figure.

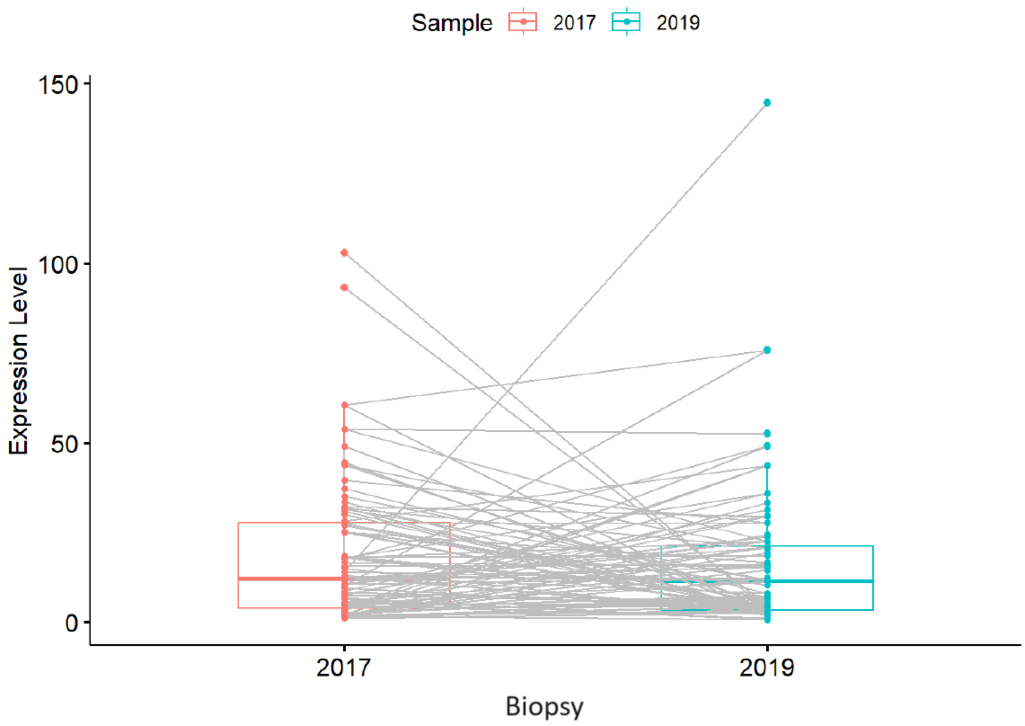

Supplementary Figure 1: Dynamics of expression of the 100 most immunogenic neoantigens in both Patient 1's pediatric-type diffuse high-grade glioma biopsies.

The table below lists the 100 most immunogenic neoantigens shared in both biopsies.

Supplementary Table 1: Top neoantigens in both Patient 1's pediatric-type diffuse high-grade glioma biopsies.

HLA allele	Neoantigen	Expression level 2017	Expression level 2019	Priority score 2017	Priority score 2019
HLA-A31:01	GSLFFLFKR	49.09523	49.02984	87	87
HLA-A31:01	SSLFFLIQR	43.9864	43.697	81	84
HLA-A02:01	SLFFLIQRV	43.9864	43.697	71	74
HLA-A31:01	KARGTDSPR	17.8204262	27.81636	66	39
HLA-A31:01	FAAPSPRWAR	17.8204262	27.81636	66	39
HLA-A31:01	SLSEKKCLR	5.24640467	3.945476	66	68
HLA-A02:01	FLFKRISSV	49.09523	49.02984	65	65

HLA allele	Neoantigen	Expression level 2017	Expression level 2019	Priority score 2017	Priority score 2019
HLA-A31:01	KLLRLVDNR	13.8691	4.50273	64	75
HLA-A02:01	FLIQRVWSL	43.9864	43.697	61	63
HLA-A02:01	GLPDPKTLLL	5.118662	7.1146	60	56
HLA-A02:01	FLLYRLPTV	0.942046038	0.633146934	60	49
HLA-A31:01	SWNSNFLFR	0.942046038	0.633146934	58	47
HLA-A31:01	AAPSPRWAR	17.8204262	27.81636	57	33
HLA-A02:01	KLHVPLPPRV	53.7786	12.2182	57	22
HLA-A02:01	TLHHRIWQA	2.46111	1.823963	56	44
HLA-A31:01	AGREGQSQR	2.227154721	4.6566874	55	37
HLA-A02:01	LLVPAGFLL	93.392331	19.158303	55	36
HLA-A31:01	NAFEHIFTR	31.71959	33.46441	54	28
HLA-A31:01	KSWVYPSIR	0.942046038	0.633146934	52	43
HLA-A02:01	SMSSGLLNL	5.31281	6.68775	51	51
HLA-A31:01	SVFRETPPR	31.9526	35.9539	50	61
HLA-A31:01	SSWTGSRASR	25.008712	20.746814	50	44
HLA-A31:01	KAHRNKIGR	11.62736	12.08026	48	48
HLA-A02:01	KLFEVTRHRM	5.52756	7.129783	47	46
HLA-A02:01	HLLIWNPPLGV	7.454681	20.7145339	47	41
HLA-A02:01	WMLPGVMTF	2.0351	2.23169	47	36
HLA-A02:01	RLDYLINV	13.94588	14.4022	47	23
HLA-A02:01	KRLYEFFNEL	18.2767	15.5721	46	43
HLA-A31:01	SSRGSKSRR	12.695568	11.224904	46	43
HLA-A02:01	RISAKLFTV	4.66696	2.64528	46	43
HLA-A02:01	RQNEHIWWL	10.60225	4.709309639	44	44
HLA-A02:01	YLKSESLSFL	4.000155658	2.66120419	44	42
HLA-A02:01	QMRFDGPLHI	5.067674	11.596717	43	28
HLA-A02:01	QLYSKVINV	39.55090248	22.31725155	43	52
HLA-A02:01	RQLYSKVINV	39.55090248	22.31725155	43	52
HLA-A31:01	LTMAEYFFR	27.4891	11.688924	43	43
HLA-A02:01	RLQPIVESV	1.31684	0.93178964	43	35
HLA-A31:01	KLHVPLPPR	53.7786	12.2182	43	16
HLA-A31:01	NQKKPFFGR	12.47263273	4.328611423	42	44
HLA-A31:01	RNQKKPFFGR	12.47263273	4.328611423	42	44
HLA-A02:01	YQHAGLMAL	18.1273	15.2668	42	41
HLA-A02:01	KLLHPKDQEVFL	12.9118	1.39511	42	39
HLA-A02:01	NMLTLPEEL	25.06	20.29875	41	48
HLA-A02:01	LIWNPPLGV	7.454681	20.7145339	41	36
HLA-A02:01	LLIWNPPLGV	7.454681	20.7145339	41	36
HLA-A31:01	TMAEYFFRK	27.4891	11.688924	41	41
HLA-A31:01	HTQALWNAR	2.0351	2.23169	41	31
HLA-A31:01	VFFDNKNSGR	30.97225	29.716343	41	34
HLA-A31:01	KSYILTNR	30.082449	31.379433	41	28
HLA-A31:01	KFGELEGTR	9.3732	15.36115	41	33
HLA-A31:01	KFEQGSHER	9.3732	15.36115	41	33
HLA-A02:01	ILASYGNAI	33.522442	29.5772255	41	42

HLA allele	Neoantigen	Expression level 2017	Expression level 2019	Priority score 2017	Priority score 2019
HLA-A31:01	AIVITINYR	33.522442	29.5772255	41	42
HLA-A02:01	LQFAESFEV	103.0627	144.8716	40	44
HLA-A31:01	NFISFRNTR	27.06509	16.35525	40	64
HLA-A02:01	SVLSGVIKI	1.59568	6.11664	40	37
HLA-A02:01	ILGWGVENV	44.49399115	52.52192001	40	29
HLA-A31:01	RVRPLPAQR	1.94556	3.17917	40	54
HLA-A31:01	ALAPAPARR	1.94556	3.17917	40	54
HLA-A31:01	AQRGRPAHR	1.94556	3.17917	40	54
HLA-A31:01	RALPPPLLVR	1.94556	3.17917	40	54
HLA-A31:01	RVRPLPAQRGR	1.94556	3.17917	40	54
HLA-A31:01	HSLPGPGRR	1.94556	3.17917	40	54
HLA-A31:01	AAHVPQTVR	1.94556	3.17917	40	54
HLA-A31:01	RAQPRPQQHR	1.94556	3.17917	40	54
HLA-A31:01	RAHPPGQLLGR	1.94556	3.17917	40	54
HLA-A02:01	TLMTEFSKL	1.977904726	2.037551074	40	44
HLA-A31:01	RTPMFPQFR	14.74243	15.857494	40	40
HLA-A31:01	ALEGKWYKR	10.60225	4.709309639	39	39
HLA-A31:01	RTMVEAAFGR	10.60225	4.709309639	39	39
HLA-A31:01	TMVEAAFGR	10.60225	4.709309639	39	39
HLA-A02:01	AVYSSTVGL	16.43635	16.60498	39	40
HLA-A31:01	KQKANSTKR	11.29083	7.272620022	39	39
HLA-A02:01	LLSSVASLV	3.35795	3.21611	39	36
HLA-A31:01	QLPGARVRR	1.94556	3.17917	39	54
HLA-A31:01	SQTNCRLSR	3.96189	2.95288	39	36
HLA-A02:01	IVSIDIPLV	3.209592	4.935202001	38	25
HLA-A31:01	YFFRKWCKR	27.4891	11.688924	38	38
HLA-A02:01	NLFLQPPQI	17.3101	23.5981	38	29
HLA-A31:01	AMMDKNADR	28.2576	18.56425	38	34
HLA-A02:01	KMPFQVEQV	6.33516	5.35319	38	24
HLA-A02:01	NLWPGDLLA	1.570771779	3.98768741	38	39
HLA-A31:01	SWTGSRASR	25.008712	20.746814	38	33
HLA-A31:01	RGQARRPRR	31.9526	35.9539	37	45
HLA-A02:01	IYLMSDSPNI	6.661101407	6.95973968	37	41
HLA-A02:01	TLAATSTAV	32.317371	24.2570553	37	29
HLA-A02:01	KLIKKQHLV	3.736280507	5.427739108	37	30
HLA-A31:01	ASCWRSRTR	25.008712	20.746814	37	33
HLA-A02:01	SQWRPVVQV	15.52077	2.885929	37	49
HLA-A31:01	RLAPFFER	37.30366232	22.27544347	37	37
HLA-A31:01	SQHPSPLKR	12.5651	10.21	37	42
HLA-A02:01	FILANHLYPV	60.41324	75.74959	37	33
HLA-A02:01	ILANHLYPV	60.41324	75.74959	37	33
HLA-A31:01	GEVMGPQIPR	9.976061	7.95685	37	39
HLA-A31:01	EVMGPQIPR	9.976061	7.95685	37	39
HLA-A02:01	SLAKNTCYL	1.97737	2.48946	36	33

1.5 Analysis of shared variants in both patients' tumors

The following figure shows shared variants in both patients' tumors identified by additional analysis of the WES data.

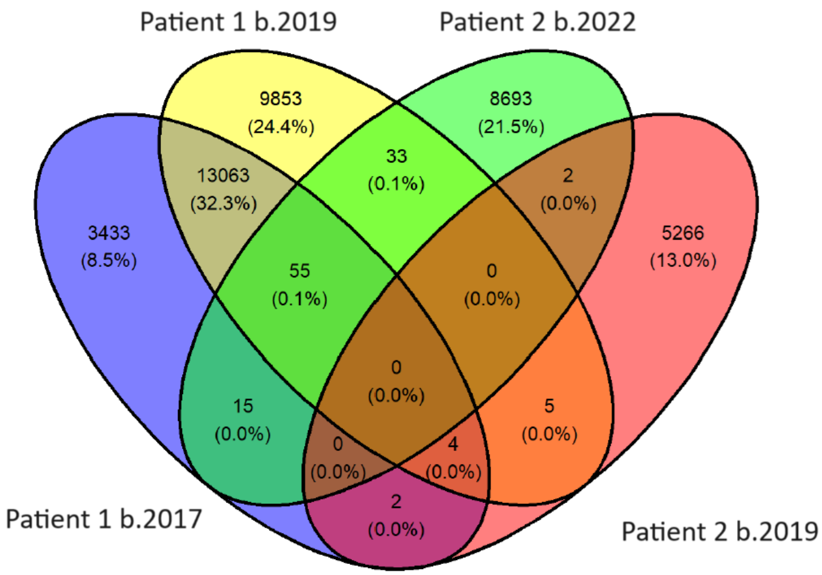

Supplementary Figure 2: Shared variants between patients and their tumors.

We can see 55 mutations shared between patients' pediatric-type diffuse high-grade glioma tumors. Only one of is considered to have a high impact on protein encoded by this gene these (splicing variant c.3+2T>C in the *ISG15* gene). None of these shared variants have been previously described or functionally characterized. Germline pathogenic mutations in the *ISG15* gene are associated with immunodeficiency (autosomal recessive inheritance) (omim.org). Its immunoregulatory role has also been studied in cancer (9) and could possibly represent a potential shared target, if such therapy was available.

2. RNA sequencing - gene expression profiling

Supplementary Table 2: Increased gene expressions of selected clinically relevant genes examined by RNA sequencing identified in Patient 1' s tumors.

**Signaling pathway**	**Gene**	**Protein**	**FC**	**Graphical representation**	**Score**
**Colorectal carcinoma**					
Receptor tyrosine kinase/growth factor signaling	*ERBB3*	Receptor tyrosine-protein kinase erbB-3	3.31	++++	1
	FGFR4	Fibroblast growth factor receptor 4	2.89	++++	1
	MET	Hepatocyte growth factor receptor	2.16	+++	1
	ROS1	Proto-oncogene tyrosine-protein kinase ROS	2.09	+++	2
	VEGFA	Vascular endothelial growth factor A	1.88	+++	1
Cell cycle control	*CCND1*	G1/S-specific cyclin-D1	2.11	+++	3
Chromatin remodeling/DNA methylation	*TERT*	Telomerase reverse transcriptase	4.45	+++++	4
**PDHGG (biopsy 2017)**					
Receptor tyrosine kinase/growth factor signaling	*PDGFRA*	Platelet-derived growth factor receptor alpha	5.33	++++++	1
	KDR	Vascular endothelial growth factor receptor 2	2.64	++++	2
	FLT4	Vascular endothelial growth factor receptor 3	2.24	++++	2
	ERBB3	Receptor tyrosine-protein kinase erbB-3	2.17	++++	2
	PDGFRB	Platelet-derived growth factor receptor beta	1.98	+++	1
Cell cycle control	*CCND1*	G1/S-specific cyclin-D1	4.66	+++++	3
	MYC	Myc proto-oncogene protein	3.54	+++++	4
**PDHGG (biopsy 2019)**					
Receptor tyrosine kinase/growth factor signaling	*VEGFA*	Vascular endothelial growth factor A	5.81	++++++	2
	PDGFRA	Platelet-derived growth factor receptor alpha	4.35	++++++	1
	ERBB3	Receptor tyrosine-protein kinase erbB-3	2.77	++++	2
	ALK	ALK tyrosine kinase receptor	2.76	++++	2
Cell cycle control	*MYC*	Myc proto-oncogene protein	2.91	++++	4
	CCND1	G1/S-specific cyclin-D1	2.86	++++	3

Score classification details (modified based on Worst *et al* (10): **Score 1**: Overexpressed driver, entity-specific; description: overexpression of a directly targetable gene known to be a driver in specific entity); example: overexpression of *PDGFR/EGFR/FGFR* etc. in HGG. **Score 2**: Overexpressed driver, entity other; description: overexpression of a directly targetable gene known to be a driver in another entity; example: overexpression of *EGFR/MET/VEGFR* etc. in an entity where this is not typically seen. **Score 3**: Pathway activation, expression, entity any; description: expression changes clearly indicating activation of a potentially actionable pathway/genes; example: MAPK pathway activation; SHH pathway activation, *CCND1*, *CDK4* overexpression. **Score 4**: *MYC*, *TERT* overexpression (not targetable, associated with cancer prognosis). High and/or aberrant Myc expression is related to poor prognosis and aggressive conditions (11). *TERT* transcription and telomerase activity contribute to cancer development and progression (12).
Abbreviations: PDHGG, pediatric-type diffuse high-grade glioma; HGG, high-grade glioma; MAPK, Mitogen-Activated Protein Kinase; SHH, Sonic Hedgehog.

Supplementary Table 3: Increased gene expressions of selected clinically relevant genes examined by RNA sequencing identified in Patient 2´s tumors.

Signaling pathway	Gene	Protein	FC	Graphical representation	Score
**HGBL-11q**					
Chromatin remodeling/DNA methylation	*TERT*	Telomerase reverse transcriptase	8.61	++++++	4
**PDHGG**					
Receptor tyrosine kinase/growth factor signaling	*PDGFRA*	Platelet-derived growth factor receptor alpha	4.74	++++++	1
	FLT4	Vascular endothelial growth factor receptor 3	2.41	++++	2
	KDR	Vascular endothelial growth factor receptor 2	1.89	++++	2
Cell cycle control	*CCND1*	G1/S-specific cyclin-D1	3.92	+++++	3
	MYC	Myc proto-oncogene protein	2.55	++++	4
	CDK4	Cyclin-dependent kinase 4	1.96	++++	3

Score classification details (modified based on Worst *et al* (10): **Score 1**: Overexpressed driver, entity-specific; description: overexpression of a directly targetable gene known to be a driver in the specific entity); example: overexpression of *PDGFR/EGFR/FGFR,* etc. in HGG. **Score 2**: Overexpressed driver, an entity other; description: overexpression of a directly targetable gene known to be a driver in another entity; example: overexpression of *EGFR/MET/VEGFR,* etc. in an entity where this is not typically seen. **Score 3**: Pathway activation, expression, entity any; description: expression changes clearly indicating activation of a potentially actionable pathway/genes; example: MAPK pathway activation; SHH pathway activation, *CCND1*, *CDK4* overexpression. **Score 4**: *MYC*, *TERT* overexpression (not targetable, associated with cancer prognosis). High and/or aberrant Myc expression is related to poor prognosis and aggressive conditions (11). *TERT* transcription and telomerase activity contributes to cancer development and progression (12).

Abbreviations: HGBL-11q, high-grade B-cell lymphoma with 11q aberrations; PDHGG, pediatric-type diffuse high-grade glioma; HGG, high-grade glioma; MAPK, Mitogen-Activated Protein Kinase; SHH, Sonic Hedgehog.

3. Microarray-based comparative genomic hybridization (array-CGH)

Supplementary Table 4: Summary of chromosomal aberrations identified by array-CGH in both patients' tumors.

Patient and sample	Chromosome	Detected aberration
Patient 1 PDHGG (biopsy 2019)	2	Loss of the whole chromosome
	8	8q22.1-q22.3 loss
	8	8q24.13-q24.21 loss
	10	10q11.21-q26.3 loss
	13	Loss of the whole chromosome
	15	Loss of the whole chromosome
	16	16q11.21-q26.3 loss
	21	Gain of the whole chromosome
	22	Loss of the whole chromosome
	X	Xq23-q28 loss
Patient 2 HGBL-11q	1	1p36.33-p32.1 LOH
	3	3q26.33-q28 gain
	3	3q28-q29 loss
	3	3q29 amplification
	7	7q11.21-q36.3 gain
	11	11q23.2-q23.3 gain
	11	11q q23.3-q25 loss
	18	18p loss
Patient 2 PDHGG	1	1q loss
	2	2p loss
	4	4p16.3-p15.31 loss
	7	7 p22.3-p21.2 loss
	7	7 p21.1-p15.3 gain
	7	7q loss
	10	10q21.1-q26.3 loss
	13	13q14.11-q34 loss
	17	17q21.33-q25.3 gain
	17	17q22-q24.1 LOH

Abbreviations: PDHGG - pediatric-type diffuse high-grade glioma; HGBL-11q - high-grade B-cell lymphoma with 11q aberrations; CGH - comparative genomic hybridization; LOH – loss of heterozygosity.

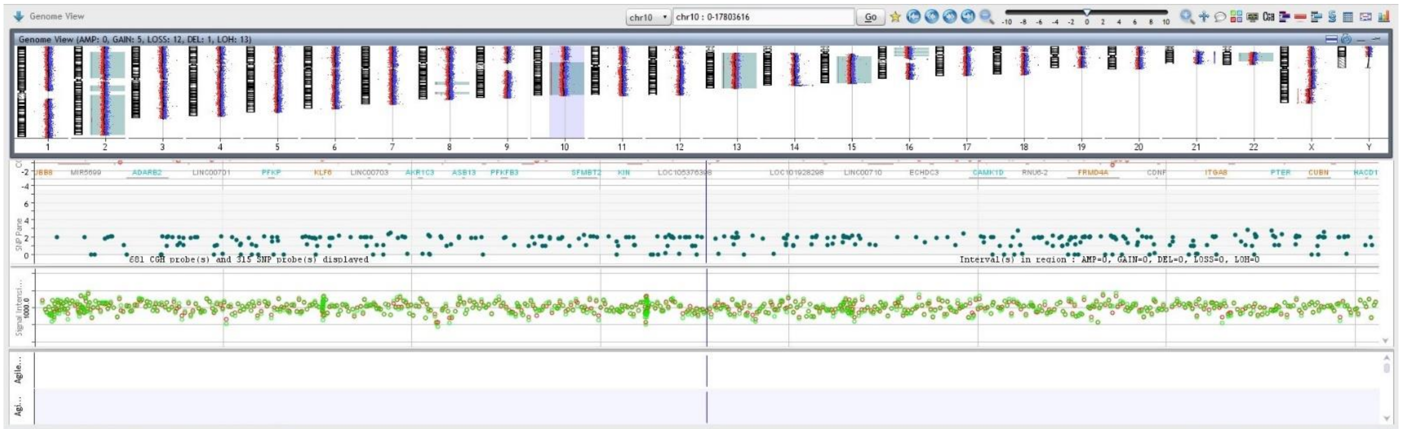

Supplementary Figure 3: Chromosomal aberrations identified in Patient 1's PDHGG (biopsy 2019) using array-CGH visualized in CytoGenomics software (Agilent).

Abbreviations: PDHGG, pediatric-type diffuse high-grade glioma; CGH, comparative genomic hybridization.

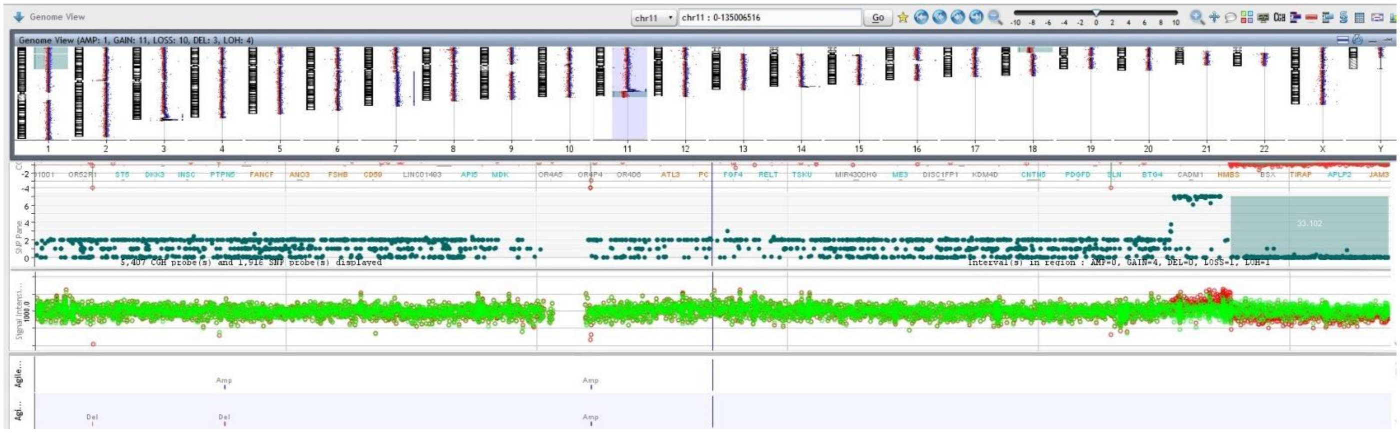

Supplementary Figure 4: Chromosomal aberrations identified in the Patient 2's HGBL-11q using array-CGH visualized in CytoGenomics software (Agilent). Aberrations affecting chromosome 11 are shown separately in Fig.2B.

Abbreviations: HGBL-11q - high-grade B-cell lymphoma with 11q aberrations; CGH - comparative genomic hybridization.

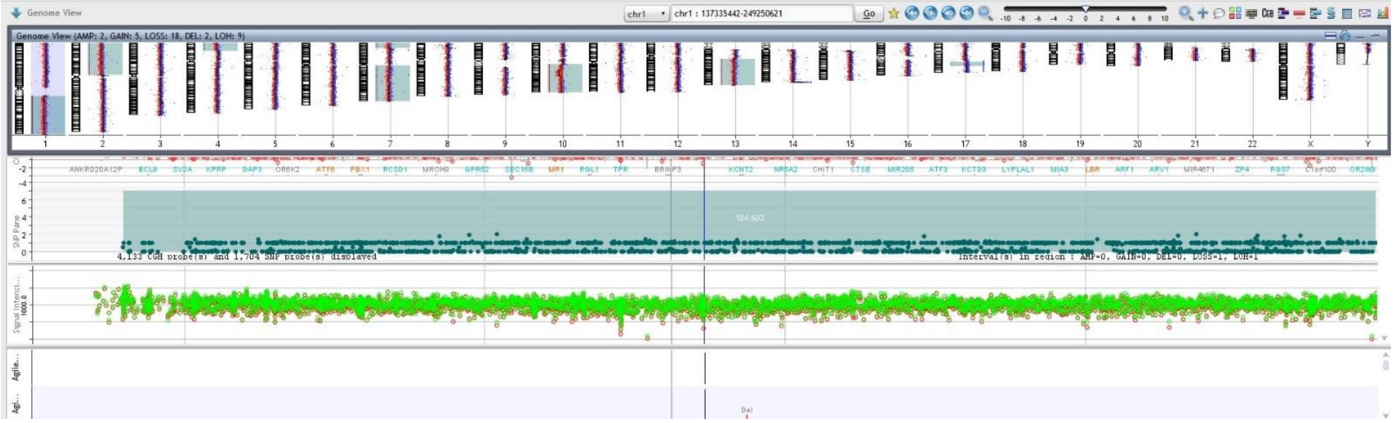

Supplementary Figure 5: Chromosomal aberrations identified in the Patient 2's PDHGG using array-CGH visualized in CytoGenomics software (Agilent).

Abbreviations: PDHGG - pediatric-type diffuse high-grade glioma; CGH - comparative genomic hybridization.

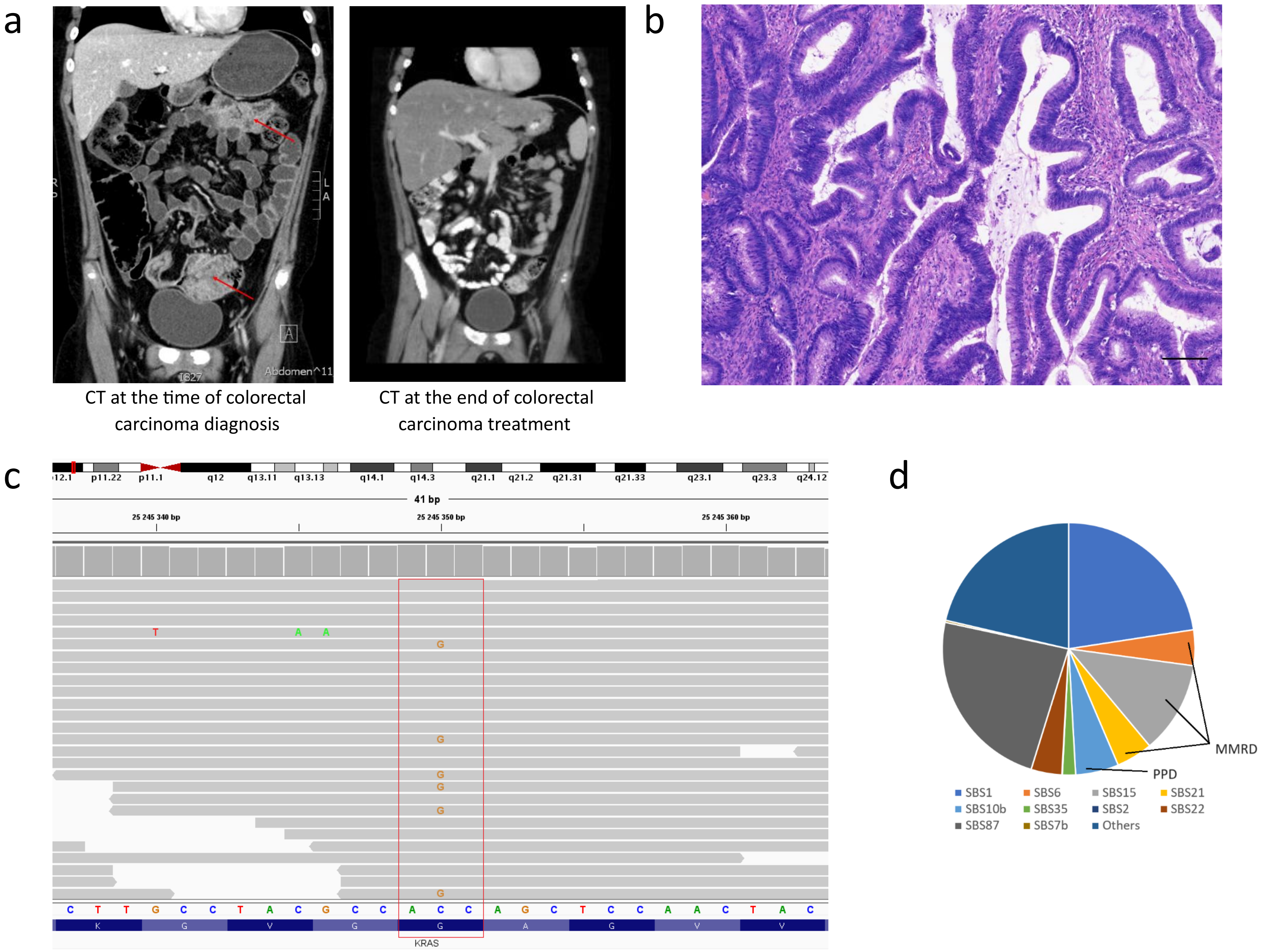
a
CT at the time of colorectal carcinoma diagnosis
CT at the end of colorectal carcinoma treatment
b
c
p11.22
p11.1
q12
q13.11
q13.13
q14.1
q14.3
q21.1
q21.2
q21.31
q21.33
q23.1
q23.3
q24.12
41 bp
25 245 340 bp
25 245 350 bp
25 245 360 bp
KRAS
d
MMRD
PPD
SBS1
SBS6
SBS15
SBS21
SBS10b
SBS35
SBS2
SBS22
SBS87
SBS7b
Others

Supplementary Figure 6: Computational tomography, histopathology, and molecular diagnostics in Patient's 1 colorectal carcinoma

a) Computational tomography (CT) at the time of diagnosis of bifocal anaplastic colorectal carcinoma and at the end of the disease.
b) Hematoxylin-eosin staining showing well-to-moderately differentiated colorectal adenocarcinoma growing into the muscularis propria. Magnification 50x, scale bar 100 µm.
c) *KRAS* c.35G>C/p.G12A mutation identified by whole exome sequencing visualized in The Integrative Genomics Viewer tool (13).
d) Mutational signatures analysis: Signatures single base substitution (SBS) 6, SBS15 and SBS21 associated with mismatch repair deficiency (MMRD) were identified in the tumor. SBS10b associated with polymerase proofreading deficiency (PPD) was also identified as the result of pathogenic *POLE* mutation.

Supplementary data references

1. Das A, Sudhaman S, Morgenstern D, Coblentz A, Chung J, et al. Genomic predictors of response to PD-1 inhibition in children with germline DNA replication repair deficiency. Nat Med. 2022 Jan;28(1):125–35.

2. Warren RL, Choe G, Freeman DJ, Castellarin M, Munro S, et al. Derivation of HLA types from shotgun sequence datasets. Genome Med. 2012;4(12):95.

3. Nariai N, Kojima K, Saito S, Mimori T, Sato Y, et al. HLA-VBSeq: accurate HLA typing at full resolution from whole-genome sequencing data. BMC Genomics. 2015;16 Suppl 2(Suppl 2):S7.

4. Iafolla MAJ, Yang C, Chandran V, Pintilie M, Li Q, et al. Predicting Toxicity and Response to Pembrolizumab Through Germline Genomic HLA Class 1 Analysis. JNCI Cancer Spectr. 2021 Feb;5(1):pkaa115.

5. Bjerregaard AM, Nielsen M, Hadrup SR, Szallasi Z, et al. MuPeXI: prediction of neo-epitopes from tumor sequencing data. Cancer Immunol Immunother. 2017 Sep;66(9):1123–30.

6. Jurtz V, Paul S, Andreatta M, Marcatili P, Peters B, et al. NetMHCpan-4.0: Improved Peptide-MHC Class I Interaction Predictions Integrating Eluted Ligand and Peptide Binding Affinity Data. J Immunol. 2017 Nov 1;199(9):3360–8.

7. Maruvka YE, Mouw KW, Karlic R, Parasuraman P, Kamburov A, et al. Analysis of somatic microsatellite indels identifies driver events in human tumors. Nat Biotechnol. 2017 Oct;35(10):951–9.

8. McLaren W, Gil L, Hunt SE, Riat HS, Ritchie GRS, et al. The Ensembl Variant Effect Predictor. Genome Biol. 2016 Jun 6;17(1):122.

9. Yuan Y, Qin H, Li H, Shi W, Bao L, et al. The Functional Roles of ISG15/ISGylation in Cancer. Molecules. 2023 Jan 31;28(3):1337.

10. Worst BC, van Tilburg CM, Balasubramanian GP, Fiesel P, Witt R, et al. Next-generation personalised medicine for high-risk paediatric cancer patients - The INFORM pilot study. Eur J Cancer. 2016 Sep;65:91–101.

11. Wang C, Zhang J, Yin J, Gan Y, Xu S, et al. Alternative approaches to target Myc for cancer treatment. Signal Transduct Target Ther. 2021 Mar 10;6(1):117.

12. Yuan X, Larsson C, Xu D. Mechanisms underlying the activation of TERT transcription and telomerase activity in human cancer: old actors and new players. Oncogene. 2019 Aug;38(34):6172–83.

13. Robinson JT, Thorvaldsdóttir H, Winckler W, Guttman M, Lander ES, et al. Integrative Genomics Viewer. Nat Biotechnol. 2011 Jan;29(1):24–6.
